# Facial Identity Recognition in the Broader Autism Phenotype

**DOI:** 10.1371/journal.pone.0012876

**Published:** 2010-09-22

**Authors:** C. Ellie Wilson, Phillipa Freeman, Jon Brock, A. Mike Burton, Romina Palermo

**Affiliations:** 1 Macquarie Centre for Cognitive Science, Macquarie University, Sydney, Australia; 2 Department of Psychology, University of Glasgow, Glasgow, United Kingdom; 3 Department of Psychology, Australian National University, Canberra, Australia; RAND Corporation, United States of America

## Abstract

**Background:**

The ‘broader autism phenotype’ (BAP) refers to the mild expression of autistic-like traits in the relatives of individuals with autism spectrum disorder (ASD). Establishing the presence of ASD traits provides insight into which traits are heritable in ASD. Here, the ability to recognise facial identity was tested in 33 parents of ASD children.

**Methodology and Results:**

In experiment 1, parents of ASD children completed the Cambridge Face Memory Test (CFMT), and a questionnaire assessing the presence of autistic personality traits. The parents, particularly the fathers, were impaired on the CFMT, but there were no associations between face recognition ability and autistic personality traits. In experiment 2, parents and probands completed equivalent versions of a simple test of face matching. On this task, the parents were not impaired relative to typically developing controls, however the proband group was impaired. Crucially, the mothers' face matching scores correlated with the probands', even when performance on an equivalent test of matching non-face stimuli was controlled for.

**Conclusions and Significance:**

Components of face recognition ability are impaired in some relatives of ASD individuals. Results suggest that face recognition skills are heritable in ASD, and genetic and environmental factors accounting for the pattern of heritability are discussed. In general, results demonstrate the importance of assessing the skill level in the proband when investigating particular characteristics of the BAP.

## Introduction

Autism spectrum disorder (ASD) is a developmental condition with widespread effects on cognitive, perceptual and motor functions. ASD is diagnosed when a triad of behavioural abnormalities are observed; specifically impaired social functioning, impaired communication, and repetitive and restricted behaviours and interests [1]. In his original description of the condition, Kanner [2] observed a number of characteristics in parents of ASD children, including “serious minded”, “mildly obsessive”, “perfectionist”, and “with an intense interest in abstract ideas”. Studies have confirmed that non-autistic relatives often exhibit characteristics of ASD in a milder, but qualitatively similar form [3]–[4]. Piven and colleagues [5]–[6] found increased rates of stereotyped behaviours, and social and communication deficits, in multiple-incidence families (i.e. those with at least two siblings with ASD). Parents of ASD children, especially the fathers, have also been reported to exhibit perceptual and cognitive characteristics of ASD, particularly a bias for processing local elements of stimuli [7]–[8]. This expression of ‘autistic-like’ traits in non-autistic relatives has come to be known as the ‘broader autism phenotype’ (BAP) [5], [9]–[10].

This study investigates facial identity recognition in the BAP. Recognising faces is an important aspect of visual processing and social functioning, facilitating appropriate interactions, and the formation of social bonds. In individuals with ASD, difficulties with facial identity recognition have been reported in a number of studies, e.g. [11]–[18] although many others found no evidence of impairment, e.g. [19]–[21]; for reviews, see [22]–[23]. Recently, it has been suggested that there is a strong heritable component to face recognition in typical development [24]–[26] as well as in developmental prosopagnosia – a condition characterized by deficient face recognition ability in the absence of acquired head injury [27]–[30]. Therefore, there is good reason to suspect that difficulties in facial identity recognition might be part of the BAP (an idea that has been previously suggested, [31]).

Previous studies have found that parents and siblings of ASD individuals show deficient processing of facial *emotion* [32]–[36]. However, only one published study has investigated facial *identity* recognition in the BAP. Dalton, Nacewicz, Johnstone, Schaefer, Gernsbacher et al. [37] tested eleven ASD children, their siblings, and twelve typically developing controls. Participants viewed photographs of personally familiar faces (family or friends), or unfamiliar faces, and were asked to decide whether or not each face was familiar to them. ASD children performed significantly below the level of the other groups. Unfortunately, siblings and controls performed at ceiling level, making their results difficult to interpret. Nevertheless, additional measures revealed intriguing group differences. Eye-movement recordings indicated that both the ASD group and their siblings fixated on the eye region significantly less than the control group (see also [32]). Furthermore, fMRI data revealed tha**t** activation of fusiform gyrus - an area of the brain involved in face processing [38] - was reduced in the ASD group *and* their siblings, compared to typically developing controls. A reduction in amygdala volume was also observed in both groups.

Our study investigated the facial recognition ability of *parents* of ASD children. We elected to study parents rather than siblings on the basis that the majority of ASD children will have one mother and one father who could be assessed, therefore providing some consistency in the sample. In order to avoid the problem of varying levels of pre-existing familiarity inherent in the use of familiar faces, we also opted to examine unfamiliar, rather than familiar, face recognition.

It is widely acknowledged that whilst some parents of ASD children may present with autistic traits, this is not necessarily the case for *all* parents of ASD children [3], [39]. Thus, in addition to considering the performance of parents as a group, we also investigated individual differences between parents. In Experiment 1, we considered the relationship between face recognition skills and degree of autistic traits. In Experiment 2, we looked at the association between parents' face recognition skills and those of their autistic children.

## Methods

### Experiment 1

Experiment 1 investigated the performance of parents on a standardized test of face recognition - the Cambridge Face Memory Test (CFMT; [40]). The CFMT is a computer-based test assessing the ability to learn and then recognize six new faces. Given the high reliability and validity of the CFMT, the test is widely used by researchers of face recognition and prosopagnosia, e.g. [41]–[45].

We also considered the relationship between parents' performance and the degree of autistic traits, measured using the Broad Autism Phenotype Questionnaire (BAPQ; [46]). The BAPQ was designed to detect the presence of three characteristics, namely aloof personality, pragmatic language impairment and rigid personality, which have been found to be more common in parents of ASD children than parents of typically developing children [6]. In a previous study, Adolphs et al. [32] reported that deficits in emotion recognition in ASD parents were most prominent in those parents that exhibited a ‘socially aloof’ personality trait. The current study extended this work to consider the relationship between distinct features of the BAP and recognition of facial identity.

#### Participants

All participants provided written informed consent to take part in this study. The research was approved by the Macquarie University human ethics committee.

Participants were the parents of 20 ASD children, who had been recruited from Macquarie University Special Education Centre, and Autism Spectrum Australia, to take part in other studies of face recognition in our lab. The children ranged in age from 7.50–12.33 years, (Mean: 9.66, SD: 1.54). All 20 children met criteria for ASD according to the DSM IV [1], and all met cut-off for an ASD on the basis of the Social Communication Questionnaire, lifetime (SCQ; [47]). Twelve of the children had been previously diagnosed using the Autism Diagnostic Interview Revised (ADI-R; [48]) or the Autism Diagnostic Observation Schedule (ADOS; [49]). A diagnosis of ASD was confirmed in the remaining eight children with the Childhood Autism Rating Scale (CARS; [50]). Fourteen of the children were classified as autistic, and six with Asperger syndrome. Where possible, both the mother and father of a child were involved. There were 13 parent pairs, six mothers (where the father did not participate) and one father (where the mother did not participate), for a total of 19 females and 14 males. The mean age of the ASD mothers was 41.26 years (SD = 5.25), and the mean age of the fathers was 44.86 years (SD = 5.86).

#### The Cambridge Face Memory Test (CFMT; [40])

Internal reliability of the CFMT is high (α = 0.89, [42]). The CFMT was presented on a 15-inch Mac Power Book laptop, following the standard instructions. The CFMT contains three phases, each with a learning and test component. In the first phase, participants view six unfamiliar male faces, cropped so that no external information, such as hair or clothing, is visible. The participants view each face, in turn, from three angles (front on, left 1/3 profile, right 1/3 profile), for 3000 ms per image. Phase 1 consists of three 3-alternative-forced-choice (3AFC) test trials for each identity, in which participants must choose which face was previously seen from two distracters (total of 18 trials). The target images are identical to those seen during learning. In phase 2, participants are shown a front view image of all six target faces simultaneously for 20 seconds to refresh their memory. In the subsequent test, participants are asked to select the target faces from a 3AFC recognition test consisting of novel images (different lighting and pose) of the six target faces and two distracters. The distracter faces were repeated on some trials, so that participants had to recognise specific target faces and not simply rely upon judgements of familiarity. There were 30 trials in this phase. In phase 3, participants were presented with another screenshot of all six target faces for 20 seconds. The recognition test was the same as that for the previous phase except that the faces were overlaid with Gaussian noise (24 trials).

The maximum overall raw score on the CFMT is 72. Given the association between age and CFMT performance [42], our analyses focus on age-standardized rather than raw scores. The age-standardized z-scores were calculated using the formula derived from a normative study of the Australian population (n = 240; [42]).

#### Broader Autism Phenotype Questionnaire (BAPQ; [46])

The BAPQ was designed to efficiently and reliably measure three personality and language characteristics that are considered to be primary components of the BAP [6]. These are ‘aloof personality’, defined as a lack of interest in or enjoyment of social interaction; problems in ‘pragmatic language’, referring to deficits in the social aspects of language; and ‘rigid personality’, which was defined as little interest in change, or difficulty adjusting to change. The items required participants to rate how frequently each statement applied to them, on a scale of 1–6. There are 12 items relating to each characteristic, or ‘subscale’ (total of 36) and 11 items were reverse scored to avoid response bias (see [40], for more details).

### Experiment 2

In Experiment 2, we considered the association between parents' face recognition skills and the face recognition skills of their children. Our own research has shown that, while children with ASD as a group are impaired on face recognition tests, many *individuals* perform at age-appropriate levels [55]; (Wilson et al., submitted). There is also increasing evidence for the heritability of face recognition skills [25]–[26] suggesting that an association may exist between the parents' performance and that of the proband.

In order to make valid comparisons between parents and probands, we devised a simple sequential face matching task based on our previous work (Wilson et al; submitted). The test has minimal memory load and is straightforward to understand, meaning that, with minor modifications, we could use the same test with the ASD children as well as their parents. Importantly, we also developed an equivalent task using photographs of shoes instead of faces, enabling us to control for potentially confounding factors such as general intellectual ability, visual processing skills, task comprehension and sustained attention. Specifically, we used control data from typically developing children and adults to calculate how well each participant performed on the faces task relative to their performance on the non-face task. Thus, we were able to directly determine the relationship between face-specific difficulties in parents and their children.

#### Participants

Participants were the same parents who had taken part in Experiment 1, together with their ASD children. One mother's data was lost due to a technical error, so her child was also removed from analyses.

#### Stimuli

For the face matching tests, 25 pairs of Caucasian young male identities were originally taken from a Glasgow Face Recognition Group database, (www.psy.gla.ac.uk/~mike/facerec.html), and had been used previously in our research (Wilson et al., submitted). For each identity there were two photographs, taken with a different camera under different lighting conditions, and transformed to greyscale. For the shoe matching test, stimuli were photographs of 20 pairs of different running shoes. As with the face task, the images were taken with two different cameras under different lighting conditions, and transformed to greyscale.

#### Face And Shoe Matching Tasks - Adult version

**Figure 1:**
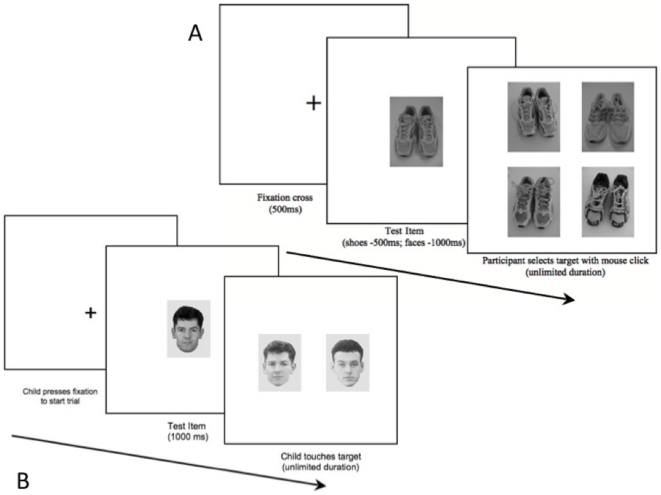
Trial Sequence. A) Adult's 4 AFC shoe matching task, B) Children's 2 AFC face matching task.

The tests were completed on a laptop (a 15” Mac, or a 32×28 cm Dell PC) using e-Prime software [56]. A fixation cross appeared in the centre of the screen for 500 ms, followed by the test item. The face test items were presented for 1000 ms, and the shoes were presented for 500 ms. Different presentation times were used in order to equate difficulty across the two tasks, because pilot tests revealed that the shoe matching task was easier than the face matching task. Following this, the target (which was a different picture of the same face/pair of shoes) and three distracters were shown, and participants responded by clicking the mouse on the selected item (e.g. Figure 1a). Each item appeared in two trials throughout the test (either once as a target and once as a distracter, or as a distracter both times). Participants completed the face test (50 trials) followed by the shoe test (50 trials). Performance on both tasks was standardized using data from 45 adults (age range 18–57 years, M = 23.94, SD = 10.46) recruited from the Macquarie Centre for Cognitive Science Paid Subjects Pool. The standardization data showed a significant correlation between performance on the face and shoe matching tasks, r (45) = 0.41, *p*<0.01. The regression equation was used to derive standardized scores for face matching based on shoe matching, cf. [57]. Importantly, although the standardization sample was younger than the ASD parents, they exhibited a similar range of performance on the two tasks. Moreover, a smaller sample of parents of non-ASD children were also tested and showed a similar relationship between scores on the two tasks. Cronbach's alpha was α = 0.61 for the adult face matching task, and α = 0.65 for the adult shoe matching task.

#### Face And Shoe Matching Tasks - Child version

Children completed a 2 AFC version of the same test, administered on a 32×28 cm touch screen monitor. Participants were told to touch the picture showing the same person/pair of shoes as the first picture. To start each trial the participant had to touch a cross that was presented in the centre of the screen, and upon release, the test item (face/pair of shoes) appeared for 1000 ms. Following this, the target and distracter appeared, and remained on screen until the participant responded by touching their selected item (e.g. Figure 1b). Distracter items were not repeated and were always novel faces. The tests included 8 practice trials, followed by 50 test trials of each type, with a break mid-way through. No feedback was given. Data from 30 typically developing children (aged 4.83–15.00 years, Mean = 9.68; SD = 2.33) were used to generate standardized scores for face matching relative to performance on the shoes task. Like the adults, their scores on the face matching task were strongly related to scores on the shoe matching tasks, r (30) = 0.68, *p*<0.001. Reliability of the tests were α = 0.73 for face matching, and α = 0.74 for shoe matching.

## Results and Discussion

### Experiment 1

In Experiment 1 we aimed to test face recognition ability in a group of parents of ASD children, and investigate associations between face recognition ability and personality traits thought to be prevalent in the BAP. At least two studies have found that BAP characteristics were more pronounced in fathers than mothers [8], [51]. In addition, it has been suggested that autistic traits are more prominent in typically developing males than females [52]. With this in mind, we analysed results from the mothers and fathers separately as well as considering the parent group as a whole.

#### CFMT

**Figure 2:**
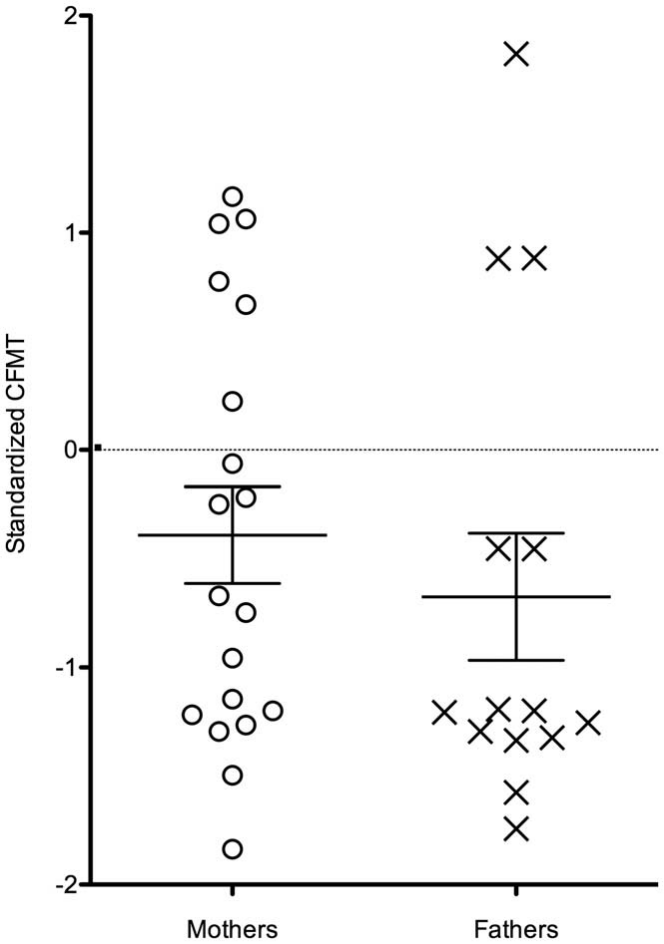
Parents' standardized CFMT scores. As a group, the fathers, but not the mothers were significantly below zero.

Mean and standard deviations of raw, and age-standardised CFMT z-scores are presented in Table 1, and age-standardized z-scores are also plotted in Figure 2. As a group, the parents' standardized scores on this test were significantly below zero, t (32) = −2.89, p<0.01. When analysed separately, however, the fathers' scores were significantly below zero, t (13) = −2.31, p = 0.04, but the mothers' scores were not, t (18) = −1.76, p = 0.10. Nevertheless, we note that none of the parents' individual scores were less than 2 SDs below the control mean, which would be indicative of severe face recognition impairments (i.e. people diagnosed with developmental prosopagnosia, cf. Bowles et al., [42]). This is consistent with the notion that autistic traits in the BAP are similar to, but milder than the equivalent trait in the proband [4].

**Table 1:** Parents' raw (%) and standardized scores, (mean, SD).

Expt	Task	Controls	Parents	Mothers	Fathers
1	**CFMT raw**	77.22 (12.92)	70.16 (12.07)	71.85 (11.37)	67.85 (13.04)
1	**Age-standardized CFMT**	n/a	−0.51 (1.02) **	−0.39 (0.97)	−0.67 (1.09) *
2	**Raw face matching**	71.72 (10.85)	72.18 (12.18)	69.20 (14.28)	76.43 (6.73)
2	**Raw shoe matching**	77.98 (8.66)	75.94 (7.91)	73.56 (6.91)	78.93 (8.61)
2	**Shoe-standardized face matching**	0 (0.99)	0.23 (1.06)	0.05 (1.24)	0.47 (0.58) ^∼^

CFMT: Parents N = 33; Mothers N = 19′; Fathers N = 14.

Face/shoe matching: Parents N = 32; Mothers N = 18; Fathers N = 14.

Scores significantly below zero, **p<0.01; * *p*<0.05.

Score significantly above zero, ^∼^ *p* = 0.01.

The CFMT raw scores for the controls are taken from Bowles et al [42] (Table 2, raw total scores for early middle age (36–49 years), N = 21). Caution is necessary when interpreting statistical comparisons with raw data because scores are not age adjusted, however one-sample t-tests suggest that both the mothers, t (18) = 2.06, *p* = 0.05, and fathers, t (13) = 2.69, *p* = 0.02, scored significantly below average.

#### BAPQ

BAPQ raw scores are provided in Table 2, and the control data from Hurley et al. [40] is provided for comparison. Within our participant sample, the fathers' mean scores were significantly higher than the mothers' on the total score, t (31) = 3.24, p<0.01, and on all the subscales (all p's<0.05). To assess the relationship between face recognition ability and BAP traits, we correlated total and subscale scores on the BAPQ with standardized scores on the CFMT. We did this for the mothers and fathers separately and for the combined parents group; however, all correlations were non-significant. In particular, the predicted association between aloof personality type, and face recognition ability was far from significant, even when mothers and fathers scores were combined, r (33) = −0.08, p = 0.65.

**Table 2:** BAPQ subscale scores for fathers and mothers (mean, SD, range).

	Mothers (N = 19)	Fathers (N = 14)	Control mean (females, N = 32; males, N = 32)	Suggested cut-off for presence of BAP traits
**Total**	2.48 (0.59) 1.42–3.61	3.19 (0.66) ** 2.06–4.42	2.74 (0.55)	3.15
**Aloof**	2.48 (0.81) 1.17–4.23	3.30 (0.86) ** 2.17–5.33	2.75 (0.78)	3.25
**Pragmatic**	2.21 (0.60) 1.17–3.58	2.88 (0.73) ** 1.67–2.27	2.45 (0.51)	2.75
**Rigid**	2.67 (0.81) 1.67–4.33	3.39 (0.76) * 2.17–4.33	3.02 (0.55)	3.5

Independent samples t-tests show fathers' mean scores are significantly higher than mothers' mean scores, *p<.05; **p<.01.

Control mean/SD scores, and the suggested cut-off scores indicating the presence of each trait, are taken from Hurley et al., [46]. Although on average the fathers from our sample scored above, and the mothers scored below the controls from the Hurley et al sample, direct comparisons were not possible because separate scores for males and females were not provided in the Hurley paper. However, Hurley et al do suggest that cut-off scores used to indicate the presence of a BAP trait will be higher in males than females, but the sample size for separate genders was too small to give reliable scores. Nevertheless, the implication is that in the general population males tend to score higher than females on the BAPQ.

This lack of association contrasts with Adolphs et al. [32] who found parents of ASD children with an aloof personality type were more impaired, and exhibited more atypical processing of emotional faces than non-aloof ASD parents. This might indicate that ‘social aloofness’ is associated with the processing of facial *emotion* rather than *identity*. The information gained from interpreting facial emotion and identity is certainly very different, and theories of face processing suggest a distinction between the processing of these two types of information [53]–[54]. Adolphs and colleagues [32] provide no indication as to how the parents' emotion recognition ability was related to the other components of the BAPQ, therefore it is unclear whether the association they report was specific to ‘aloofness’. A study including a larger sample of BAP parents completing both facial identity and emotion recognition tasks could address this possibility.

### Experiment 2

The results of Experiment 1 confirmed that some parents of children with ASD have difficulty on tests of facial identity recognition, but found no associations with severity of autistic traits as measured by the BAPQ. In Experiment 2, we tested face recognition skills of parents and probands using a simpler test of face matching. Raw scores on the face and shoe matching tasks, and standardized face matching scores are provided in Table 1. Parents' standardized scores on the face task correlated with standardized scores on the CFMT, r (33) = 0.40, *p* = 0.03. In contrast to the CFMT, the parents achieved standardized scores that were not below normal on this task; in fact the fathers mean score was significantly greater than zero, t (13) = 3.02, *p* = 0.01.

This discrepancy with the CFMT might be accounted for by the different methods of standardization (standardized for age in the CFMT vs. standardized for shoe matching performance in the face matching test). However, face matching raw scores were also comparable for ASD parents and controls on the matching task (all *p*'s>0.7), which contrasts with the CFMT where raw scores of ASD parents were below raw scores of the control data (Table 1).

The ASD children's mean standardized score was −0.80 (SD = 1.42), which was significantly below zero, t (19) = −2.45, *p* = 0.03. This was in line with the majority of previous studies showing impaired face recognition in ASD, e.g. [11], [14], [16]. However, consistent with our own previous work (Wilson et al., submitted), the performance of the children with ASD was highly variable.

**Figure 3:**
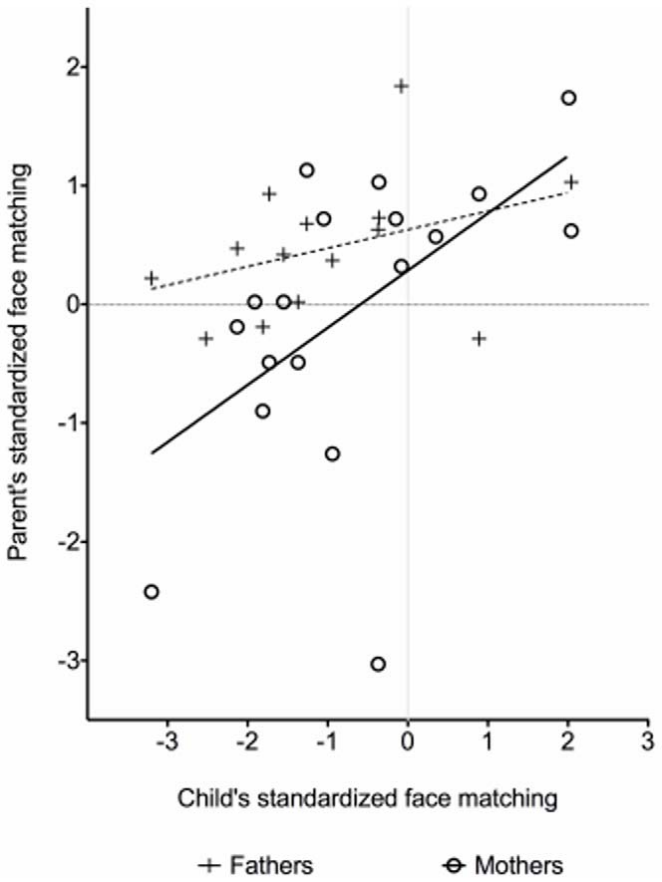
Parent and probands' standardized performance on the face matching task.

Correlational analyses revealed a positive, but non-significant relationship between the probands' face recognition scores and fathers' scores, r (14) = 0.37, *p* = 0.12, and a significant positive relationship with mothers' scores, r (18) = 0.54, *p* = 0.02 (Figure 3). Importantly, our tasks ensured that general aspects of intelligence and visual perceptual ability were controlled for, and therefore could not be responsible for the association. As in Experiment 1, there was no significant association between parents' face matching scores and their BAP traits as measured by the BAPQ. Thus, the results of Experiment 2 suggest that specific face recognition difficulties may be a heritable component of the BAP that is distinct from general ability.

### General Discussion

The current study set out to answer two questions. First, given evidence of face recognition difficulties in individuals with ASD, we aimed to determine whether similar but perhaps milder deficits could be identified in relatives of children with ASD. In some respects, the results here were somewhat contradictory. In Experiment 1, we found that parents were significantly impaired on the CFMT. However, we failed to find a significant deficit on another simpler sequential face matching task.

The discrepancy between the tasks might be accounted for by the different procedures used for standardization: performance on the sequential matching task was standardized against performance on a matched control task, whereas the CFMT was standardized against age. Thus, it could be argued that the standardized scores on the face matching task were normal because the ASD parents were performing poorly on both face matching and the control task; however the raw scores were also normal. Alternatively, differing task requirements might account for the discrepancy. For instance, the CFMT is a complex task, emphasising longer-term memory and learning of a small number of faces. By contrast, the sequential matching task is relatively simple, and tests immediate recognition of a larger number of different faces. In addition, the CFMT presents the same distracter faces several times (whereas the matching task presents the same distracter a maximum of two times for adults) thus requiring participants to recognise specific targets, rather than making a familiarity judgment. Thus, it may be that the CFMT is more tuned to the difficulties faced by the parents, particularly the fathers, of ASD children. In support of this we note that people who report severe everyday face difficulties (i.e., developmental prosopagnosics) are more likely to show poor performance on the CFMT than other more perceptual tests, like the Cambridge Face Perception Test and the Glasgow Face Perception Test [42]. However, we note that whilst the two tasks are likely tapping into different aspects of face processing, the significant association between standardized scores suggests that there is a common function - recognition of faces - that they both assess.

The second key aim of this study was to examine what factors were associated with parents' facial identity recognition ability. We found no significant association with BAP traits as measured by the BAPQ [46] in either experiment. However, we did find that children's performance on the sequential matching task was significantly associated with performance of the mothers. This alignment of skill level across related individuals suggests a hereditary basis to face recognition may exist in ASD. This proposal is consistent with two recent studies with large samples of typically developing adult twins that have found evidence for a genetic factor underlying face recognition where performance on face recognition tests were correlated more closely in monozygotic than dizygotic twin pairs [25]–[26]. These studies are also in line with several studies suggesting that developmental prosopagnosia runs in families [27]–[30]. Despite the strong genetic contribution to face recognition, the twin studies, not surprisingly, also show that there is a contribution of the environment [24].

The finding that it was the *mothers* that appeared to be driving the association was somewhat unexpected since it is typically the fathers that are thought to exhibit stronger BAP characteristics, e.g. [8], [51], and indeed the fathers that had more difficulty on the CFMT. First, we note that the sample of fathers was smaller than the mothers and there was a trend towards an association, therefore the lack of significance might have been due to a lack of power. Nevertheless, we consider two potential explanations for this asymmetrical influence of parents. At the genetic level, a process of ‘imprinting’ involves certain genes being marked for expression from either the mother or the father [58]–[60]. If face recognition ability is an independently heritable skill, it is possible that the gene responsible is subject to imprinting, and would therefore exhibit a consistent maternal or paternal bias of expression.

Alternatively, environmental factors could be responsible for the increased influence of the mothers. Typically, mothers spend more time in direct contact with their young children than fathers do, and the mother's tendency to engage her child in face to face contact, and to direct her child's attention to face stimuli in the environment might be dictated by her own skill level in this domain. This in turn may affect the child's experience of faces, thus moderating the development of skills for accurate face processing, cf. [61].

These findings warrant further research, using a variety of tests assessing different aspects of face recognition, in individuals of both typical and atypical development. Our results support the hypothesis of a familial basis to face recognition in ASD, which fits well with previous literature from other populations, but requires replication with far larger samples. Interestingly, in our previous work with developmental prosopagnosia, we found that out of four childhood cases, three had *mothers* with face recognition deficits, while in the fourth child there was no evidence of a familial basis [62].

Finally, our results also have more general implications for researching symptoms that occur in the BAP. It is widely accepted that symptom profiles in ASD are heterogeneous, therefore it is unreasonable to expect that consistent patterns of impairment would be present across relatives of ASD individuals. Our results suggest that, to effectively identify cognitive and behavioural traits that translate to the relatives of ASD individuals, it is important to take account of the corresponding trait in the ASD proband.
